# Comparative Analysis of Microbial Communities in Each Developmental Stage of *Dermacentor nuttalli* in Two Regions in Inner Mongolia, China

**DOI:** 10.3390/biology14060613

**Published:** 2025-05-27

**Authors:** Li Zhao, Xiao-Nan Dong, Hao Cui, Lian-Yang Sun, Ren Mu, Ming Nie, Jia-Mei Kang, Nan Bu, Yi-Shuai Zhang, Ze-Hao Qi, Zi-Xuan Li, Zi-Long Zhang, Xu-Yang Zhang, Yu-Lin Ding, Rui Wang, Yu Wang, Yong-Hong Liu

**Affiliations:** 1 College of Veterinary Medicine, Inner Mongolia Agricultural University, Hohhot 010018, China; zhaolidky@126.com (L.Z.); dxn1005@126.com (X.-N.D.); cuihao460009396@126.com (H.C.); 18748237178@163.com (L.-Y.S.); kjm151476@163.com (J.-M.K.); bunansyxy@163.com (N.B.); zyssyxy@163.com (Y.-S.Z.); qzhsyxy@163.com (Z.-H.Q.); lzxsyxy@163.com (Z.-X.L.); 123zzl.z@163.com (Z.-L.Z.); zxyyxz666@163.com (X.-Y.Z.); dingyulin2001@126.com (Y.-L.D.); wr2006@163.com (R.W.); 2 Key Laboratory of Clinical Diagnosis and Treatment Technology in Animal Disease, Ministry of Agriculture and Rural Affairs, Hohhot 010011, China; 3 College of Life Science and Technology, Inner Mongolia Normal University, Hohhot 010022, China; murensk@imnu.edu.cn; 4 Alxa Left Banner Animal Disease Prevention and Control Center, Alxa League 750300, China; nieming0314@163.com

**Keywords:** *Dermacentor nuttalli*, Inner Mongolia, microbial population, viral metagenomics

## Abstract

*Dermacentor* is the most widely distributed tick genus in China. *Dermacentor nuttalli*, a predominant tick species in Inner Mongolia, can carry and transmit pathogenic microorganisms. Here, *D. nuttalli* were collected from Ordos (O-D) and Hinggan League (H-D) in the Inner Mongolia. *D. nuttalli* specimens at different developmental stages were subsequently reared under identical laboratory conditions. Sample processing, nucleic acid extraction, high-throughput sequencing, and microbial community analyses were conducted. Bacterial communities in O-D and H-D were annotated to 8 phyla, 145 genera and 16 phyla, 141 genera, respectively, with Proteobacteria showing the highest relative abundance. Differences in dominant bacterial genera were observed across developmental stages between the two regions. The most abundant bacterial species were *Arsenophonus*_uncultured_bacterium in O-D and *Rickettsia japonica* in H-D. Viral communities were annotated to 4 orders, 25 families, 61 genera, and 126 species in O-D and 6 orders, 28 families, 49 genera, 135 species in H-D. Notable difference in the viral genera with >1% abundance were identified at different developmental stages in the two regions. To our knowledge, this is the first study to compare microbial community compositions of *D. nuttalli* across developmental stages in two Inner Mongolian regions under under identical rearing conditions and to report the presence of *R. japonica*, Tacheng Tick Virus-2, and bovine viral diarrhea virus in *D. nuttalli*.

## Simple Summary

In this study, nucleic acids were extracted from *Dermacentor nuttalli* collected from Ordos and Hinggan League in the Inner Mongolia Autonomous Region. Subsequently, different developmental stages of *D. nuttalli* were artificially fed under controlled laboratory conditions. Then, microbial community structure analysis was conducted using high-throughput sequencing. We first compared the microbial compositions of different developmental stages of *D. nuttalli* from the tow regions of Inner Mongolia under identical artificial feeding conditions, and annotated *Rickettsia japonica*, Tacheng tick virus 2, and bovine viral diarrhea virus in *D. nuttalli* for the first time.

## 1. Introduction

Ticks are obligatory blood-feeding arthropods that parasitize various vertebrates, including wild animals, livestock, and humans [1]. There are 949 tick species worldwide, belonging to four families—Ixodidae, Argasidae, Nuttalliellidae, and Deinocrotonidae—of which 731 species belong to Ixodidae [2]. In total, 124 species of ticks (113 hard ticks and 11 soft ticks) are found in China [3]. *Dermacentor nuttalli*, a hard tick, has been recorded in at least 11 provinces in China [4]. Its distribution range in mainland China is second only to that of *Haemaphysalis longicornis*, and it is the dominant tick species in Inner Mongolia [3,5].

Ticks transmit a remarkable diversity of pathogens, and the range of infection sources they carry exceeds that of other blood-feeding arthropods [6]. In China, *D. nuttalli* has been identified as a vector of various pathogenic microorganisms in the provinces of Inner Mongolia, Xinjiang, Yunnan, Gansu, Shaanxi, Shanxi, Heilongjiang, and Jilin, as well as in neighboring countries such as Mongolia and Russia [3]. Previous studies have reported the detection of adult *D. nuttalli* ticks [7,8,9,10,11,12,13,14,15,16,17,18,19,20,21,22,23,24,25,26,27,28] and *D. nuttalli* at different developmental stages carrying specific pathogens [29,30,31,32,33,34]. Moreover, pathogens have been annotated in adult *D. nuttalli* ticks [35,36,37,38,39,40,41,42,43] and in ticks at different developmental stages using next-generation sequencing [44]. These findings adequately demonstrate the strong ability of *D. nuttalli* to harbor and transmit pathogenic microorganisms.

High-throughput sequencing enables rapid and efficient analysis of microbial communities without the need for bacterial culture, allowing simultaneous identification of nonculturable bacteria and unknown pathogens [44,45,46]. Moreover, different tick species have preferences for specific biotopes or environments, which in turn determine their geographical distribution. Ticks with different geographical distributions may carry and transmit different pathogenic species [44]. In this study, *D. nuttalli* collected from southwestern and northeastern Inner Mongolia, regions separated by >1500 km, were artificially fed in the laboratory. High-throughput sequencing was performed using 16S rDNA amplicons and viral metagenomics techniques. We compared the microbial community structures of *D. nuttalli* at different developmental stages in the two regions, aiming to provide a scientific basis for risk assessment of tick-borne diseases.

## 2. Materials and Methods

### 2.1. Study Area and Sample Collection

**Figure 1:**
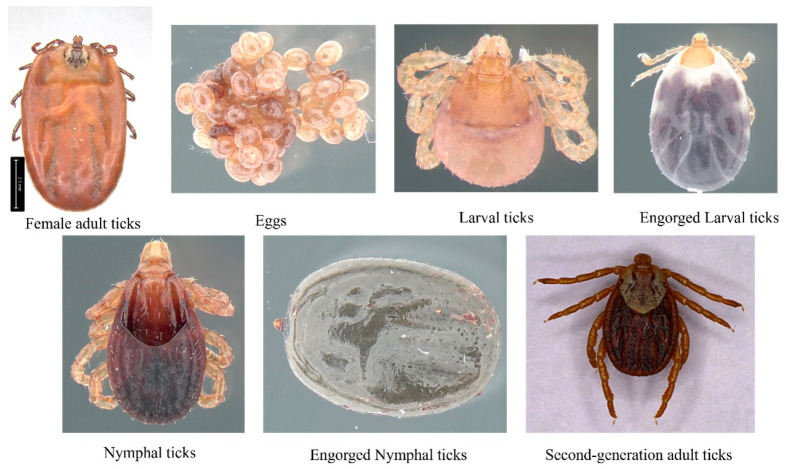
Different developmental stages of *Dermacentor nuttalli*.

A large number of adult *D. nuttalli* ticks were collected from sheep in Ordos (950 m above sea level; 109.27°′ E, 40.25° N) and cattle in the Hinggan League (950 m above sea level; 120.57°′ E, 46.57° N) in Inner Mongolia, China. Adult ticks were identified as *D. nuttalli* (designated as O-D and H-D, respectively) using morphological and molecular biological identification methods [44]. The remaining six developmental stages of *D. nuttalli* (O-D-E and H-D-E: eggs; O-D-L and H-D-L: larvae; O-D-EL and H-D-EL: engorged larvae; O-D-N and H-D-N: nymphs; O-D-EN and H-D-EN: engorged nymphs; and O-D-SA and H-D-SA: second-generation adults) were obtained by artificially rearing the first-generation adult female ticks (O-D-FA and H-D-FA) collected from the field (Figure 1). Male Kunming mice (age: 6–8 weeks; body weight: 28–32 g), purchased from Sibefu (Beijing) Biotechnology Co., Ltd. (license number : SYXK [Meng] 2020-0003), were used as the sole blood source for tick feeding. All samples were stored at −80 °C until further analysis.

### 2.2. DNA and RNA Extraction

The 14 samples represented ticks at seven developmental stages from the two regions. For each region, a well-developed tick breeding series was selected, in which six developmental stages were the offspring of an adult female tick. Each developmental stage was a pool comprising varying numbers of individual ticks. The samples were washed with sterile water followed by 75% ethanol, and DNA was extracted using the TaKaRa Mini BEST Universal Genomic DNA Extraction Kit Ver.5.0 (Takara, Beijing, China). For RNA and viral nucleic acid extraction, six mixed sample pools were prepared from the O-D and H-D groups. The pools included O-D-FA-MIX and H-D-FA-MIX (pooled first-generation adult female ticks); O-D-EL-MIX and H-D-EL-MIX (mixed samples of eggs and larvae); and O-D-SA-MIX and H-D-SA-MIX (second-generation adult ticks). All sample pools were washed with sterile water and 75% ethanol prior to nucleic acid extraction using the RaPure Viral RNA/DNA Kit (MAGEN, Shanghai, China). The extracted nucleic acids were then stored at −80 °C until further use.

### 2.3. Library Construction and High-Throughput Sequencing

The 16S rDNA V3−V4 region was amplified by using TransStart R^©^ FastPfu DNA polymerase (TransStart, Beijing, China) from the DNA of each sample. The primers 338F (5′-ACTCCTRCGGGAGGCAGCAG-3′) and 806R (5′-GGACTACCVGGGTATCTAAT-3′) were used under the following conditions: 95 °C for 2 min, followed by 25 cycles at 95 °C for 30 s, 55 °C for 30 s, and 72 °C for 30 s with a final extension at 72 °C for 5 min [47]. The PCR products were identified via electrophoresis and recovered using the AxyPrep DNA gel extraction kit (AXYGEN, Suzhou, China). Subsequently, the VAHTS ^®^ ssDNA Library Prep Kit (Illumina, San Diego, CA, USA) was used to construct the Illumina PE250 library. High-throughput sequencing of 16S rRNA was performed on the Illumina Novaseq 6000 platform (San Diego, CA, USA).

After determining the RNA concentration, the RNA samples were fragmented, followed by the addition of first strand buffer and first strand enzyme for cDNA synthesis. End repair was performed using SEA Enzyme Mix and SEA Buffer. The Fast RNA-Seq Library Prep Kit (Illumina, San Diago, CA, USA) was used to construct the Illumina PE150 library. Viral metagenomic sequencing was conducted on the Illumina NovaSeq 6000 platform (San Diego, CA, USA).

### 2.4. Data Analysis

The Illumina PE250 sequencing data were first demultiplexed based on barcode information to obtain valid sequences., Paired-end reads of the original DNA fragments were merged using FLASH (v.1.2.11), and sequence analysis was conducted using USEARCH (v.7.0.1090, http://drive5.com/uparse/) (accessed on 20 April 2024). Sequences with ≥97% similarity were clustered into operational taxonomic units (OTUs), and taxonomic classification was performed using the RDP Classifier (v.2.2, http://sourceforge.net/projects/rdp-classifier/) (accessed on 20 April 2024), based on a Bayesian algorithm. Alpha diversity analysis was conducted to evaluate community richness and diversity. Richness was assessed using the number of unique OTUs per sample, the Chao index (http://www.mothur.org/wiki/Chao) (accessed on 20 April 2024), and the abundance-based coverage estimator (ACE). Diversity was accessed using the Shannon index (http://www.mothur.org/wiki/Shannon) (accessed on 20 April 2024), and Simpson index (http://www.mothur.org/wiki/Simpson) (accessed on 20 April 2024), whereas sequencing depth was evaluated using Good’s coverage (http://www.mothur.org/wiki/Coverage) (accessed on 20 April 2024). Species accumulation curves were used to assess sample community diversity and estimate species richness.

The Illumina PE150 sequencing data were first optimized and quality-controlled using Cutadapt (v.1.16, https://cutadapt.readthedocs.io/en/stable/) (accessed on 20 April 2024) and FastQC (v.0.11.4, https://www.bioinformatics.babraham.ac.uk/projects/fastqc/) (accessed on 20 April 2024). High-quality sequences were then assembled using MEGAHIT (v.1.2.9, https://github.com/voutcn/megahit) (accessed on 20 April 2024) and gene prediction was performed with MetaGeneMark (v.3.38, http://exon.gatech.edu/GeneMark/) (accessed on 20 April 2024). Gene annotation was conducted using DIAMOND (https://github.com/bbuchfink/diamond) (accessed on 20 April 2024).

Sequencing and sequence analysis were conducted in collaboration with Origin-gene Biology Co., Ltd. (Shanghai, China).

## 3. Results

### 3.1. Sequencing Results of Illumina PE250

#### 3.1.1. General Statistics

Fourteen samples were sequenced using the Illumina PE250 platform. The O-D and H-D groups yielded 1,191,556 and 954,312 raw reads, respectively. After optimization, 562,680 and 435,060 high-quality sequences were obtained. The total number of bases in the optimized data was 237,005,186 bp for O-D and 183,324,532 bp for H-D. The average sequences lengths were 421.20 bp and 421.38 bp, respectively (Table 1).

#### 3.1.2. Alpha Diversity Analysis

**Figure 2:**
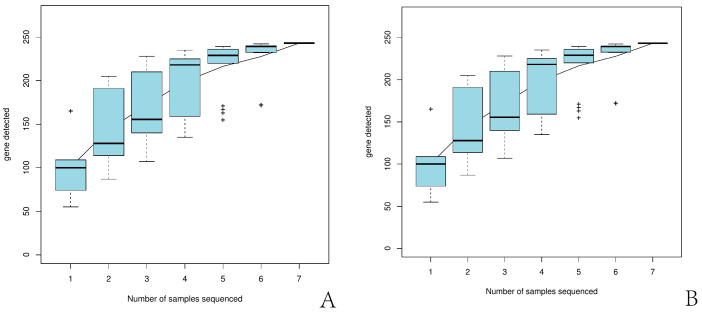
Species accumulation curves of *Dermacentor nuttalli* from the two regions: (**A**) shows the species accumulation curve for *D. nuttalli* from Ordos, and (**B**) shows the curve for *D. nuttalli* from Hinggan League. + represents outlier.

Based on the 97% similarity threshold, the Shannon, Chao, and ACE indices are presented in Table 2. The Good’s coverage of all O-D and H-D samples exceeded 99.9% (Table 2). The species accumulation curves for O-D and H-D (Figure 2) showed a tendency to plateau as sample size increased.

#### 3.1.3. OTU Cluster Analysis

**Figure 3:**
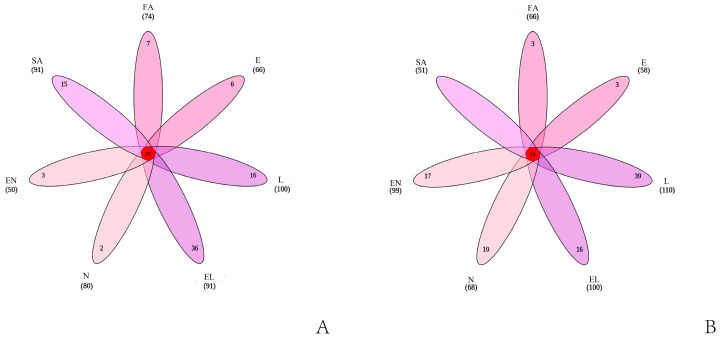
Venn diagrams of microbial OTUs identified in *Dermacentor nuttalli* from the two regions: (**A**) shows the Venn diagram of *D. nuttalli* from Ordos, and (**B**) shows that of *D. nuttalli* from Hinggan League.

A total of 660 and 620 OTUs were obtained from O-D and H-D, respectively, with 20 unique OTUs shared across all developmental stages. Each developmental stage of O-D contained unique OTUs, whereas H-D-SA did not have any unique OTUs (Figure 3). In total, 99.13% and 99.95% of tags from O-D and H-D, respectively, were assigned to the genus level. Among these, 49.88% of O-D tags and 73.38% of H-D tags were further classified at the species level.

### 3.2. Microbial Population

#### 3.2.1. Microbial Community Composition at the Phylum Level

**Figure 4:**
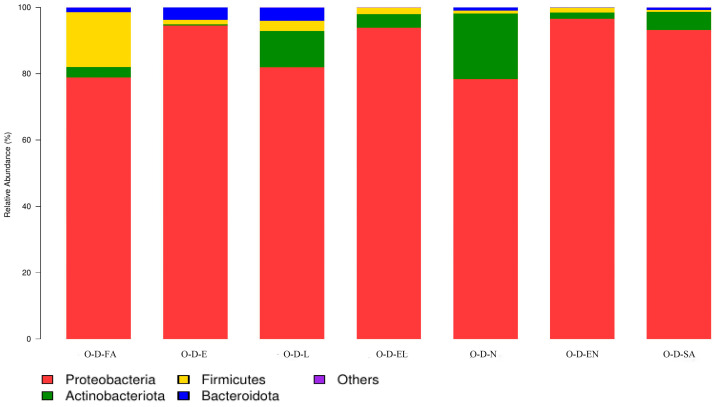
Microbial community composition at the phylum level in each developmental stage of *Dermacentor nuttalli* from Ordos. (Note: To enhance visualization, taxa with relative abundance <1% are grouped as “Others” in the graph).

**Figure 5:**
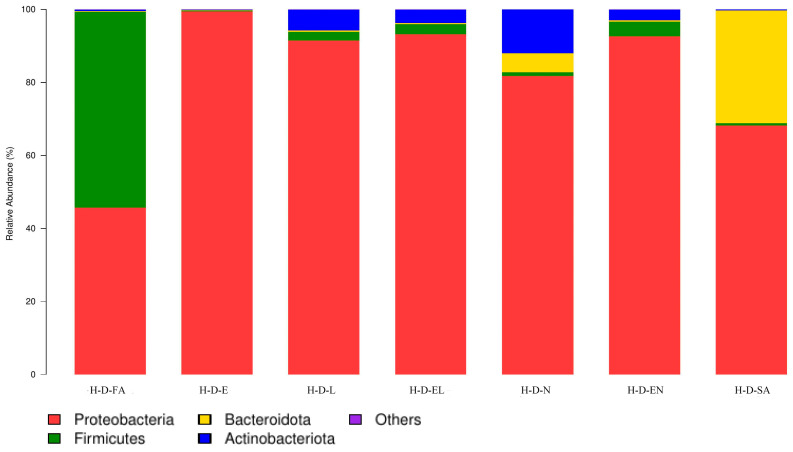
Microbial community composition at the phylum level in each developmental stage of *Dermacentor nuttalli* from Hinggan League. (Note: To enhance visualization, taxa with relative abundance <1% are grouped as “Others” in the graph).

O-D and H-D were co-annotated to eight and sixteen bacterial phyla, respectively. Across all developmental stages in both regions, four common phyla were consistently identified (Figure 4 and Figure 5). *Proteobacteria* was the dominant phylum in all developmental stages of O-D and also predominated in H-D, except in H-D-FA, where *Firmicutes* was more abundant. *Proteobacteria* had the highest total relative abundance among bacterial phyla in both regions—88.1% in O-D and 81.7% in H-D (Table 3).

#### 3.2.2. Microbial Community Composition at the Genus Level

**Figure 6:**
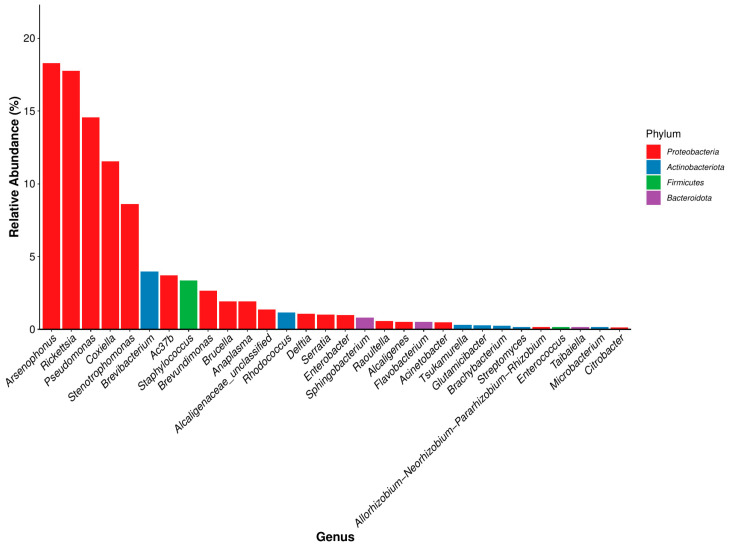
Dominant bacterial genera in *Dermacentor nuttalli* from Ordos.

**Figure 7:**
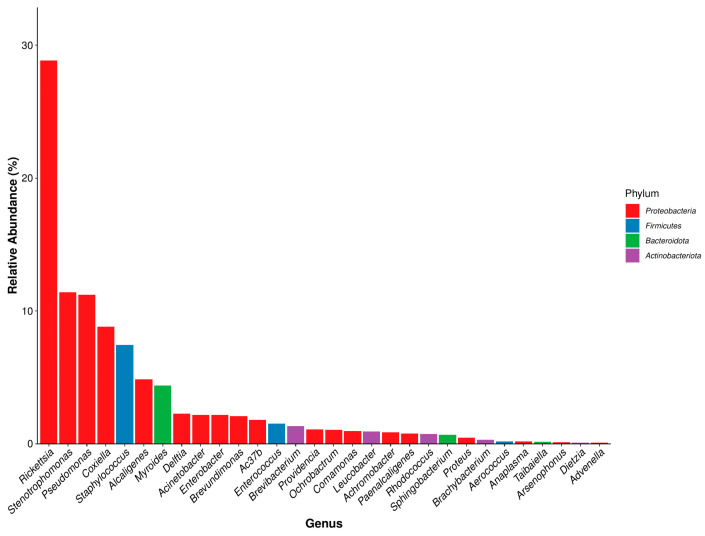
Dominant bacterial genera in *Dermacentor nuttalli* from Hinggan League.

O-D and H-D samples were co-annotated to 145 and 141 bacterial genera, respectively. The most abundant genera overall were *Arsenophonus* (18.31%) and *Rickettsia* (28.83%). In O-D, 18 of the 30 most abundant genera belonged to *Proteobacteria*, whereas in H-D, 19 were classified under *Proteobacteria*. In total, 15 and 16 genera had relative abundances greater than 1% in O-D and H-D, respectively. Across the seven developmental stages, 19 genera were annotated in O-D and 16 in H-D. The top five most abundant genera in O-D and the top four in H-D all belonged to *Proteobacteria* (Figure 6 and Figure 7). The dominant genera varied across developmental stages and between the two regions (Table S1).

#### 3.2.3. Microbial Community Composition at the Species Level

O-D and H-D were co-annotated to 28 and 36 bacterial species with an abundance of >1%, 16 and 19 bacterial species were present at seven developmental stages, respectively. The dominant species of O-D-FA, H-D-FA, O-D-E, H-D-E, and H-D-EL was *Rickettsia japonica*. The dominant species of the remaining nine samples were different (Table S2).

### 3.3. Illumina PE150 Sequencing Results

#### 3.3.1. Species Classification Annotation Data

In the three O-D samples, 74,766,240; 63,544,512; and 59,774,580 clean reads were obtained, with CG contents of 57.31%, 54.13%, and 53.69%, respectively. Following open reading frame (ORF) prediction, 342,780; 253,560; and 640,677 reads were annotated as viral sequences. For the three H-D samples, 31,468,902; 55,601,958; and 52,845,094 clean reads were obtained, with CG contents of 55.25%, 53.86%, and 54.54%, respectively. Following ORF prediction, 123,987; 23,898; and 2,851,584 reads were annotated as viral sequences.

#### 3.3.2. Species Composition at the Order Level

O-D and H-D were annotated to four and six viral orders, respectively. Among them, four viral orders, including Caudovirales, Herpesvirales, Ortervirales, and norank_d_Viruses, co-existed in all six samples (Table 4).

#### 3.3.3. Species Composition at the Family Level

**Figure 8:**
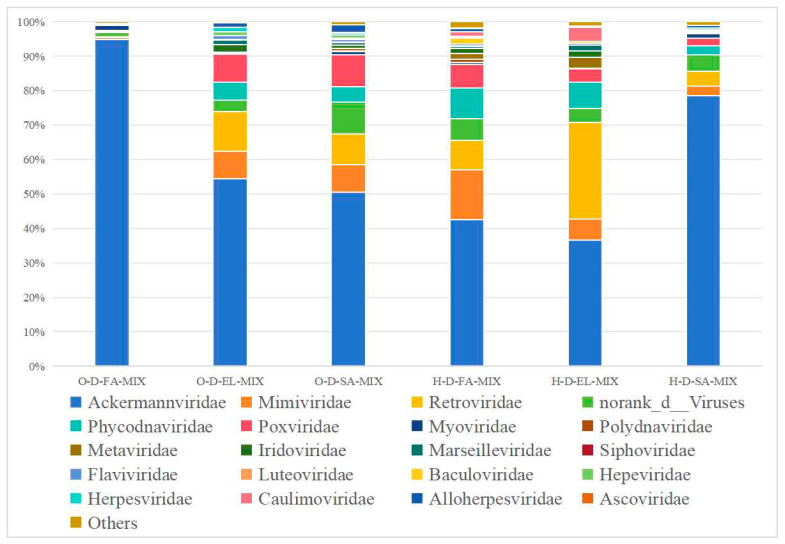
Species composition distribution at the family level. (Note: To enhance visualization, taxa with relative abundance <1% are grouped as “Others” in the graph).

O-D and H-D samples were annotated to 25 and 28 viral families, respectively (Figure 8). Among them, three pooled samples from each region shared 16 and 13 viral families, respectively. Additionally, O-D-FA-MIX and O-D-EL-MIX were co-annotated with five viral families, namely, Ascoviridae, Baculoviridae, Caulimoviridae, Metaviridae, and Polydnaviridae; H-D-EL-MIX and H-D-SA-MIX were also co-annotated with seven viral families, namely, Asfarviridae, Caulimoviridae, Geminiviridae, Iflaviridae, Marseilleviridae, Metaviridae, and Polydnaviridae; H-D-FA-MIX and H-D-SA-MIX were co-annotated with five viral families, namely, Ascoviridae, Hepeviridae, Phenuiviridae, Siphoviridae, and norank_o_Caudovirales; and O-D-FA-MIX and O-D-SA-MIX were co-annotated with norank_o_Caudovirales.

#### 3.3.4. Species Composition at the Genus Level

**Figure 9:**
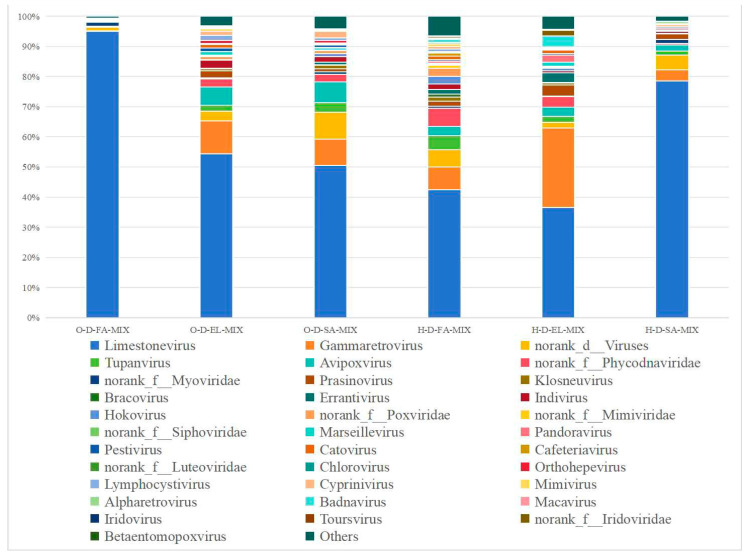
Species composition distribution at the genus level. (Note: To enhance visualization, taxa with relative abundance <1% are grouped as “Others” in the graph).

O-D and H-D samples were annotated to 61 and 49 viral genera, respectively (Figure 9, Table S3). Among them, three pooled samples from each region shared 35 genera in O-D and 23 genera in H-D. Additionally, O-D-FA-MIX and O-D-EL-MIX were co-annotated with 7 genera; O-D-FA-MIX and O-D-SA-MIX were co-annotated with Phaeovirus and norank _o_Caudovirales; O-D-EL-MIX and O-D-SA-MIX were co-annotated with 4 genera; H-D-FA-MIX and H-D-SA-MIX were co-annotated with 12 genera; and H-D-EL-MIX and H-D-SA-MIX were co-annotated with 19 genera.

#### 3.3.5. Species Composition at the Species Level

**Figure 10:**
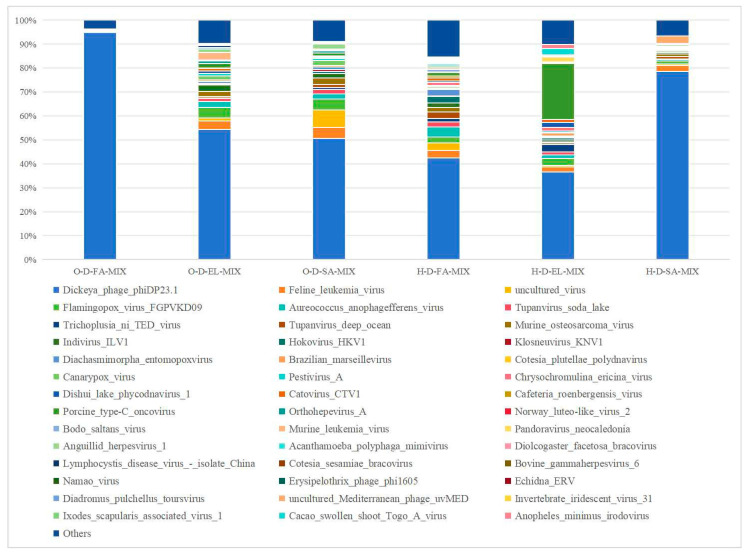
Species composition distribution at the species level. (Note: To enhance visualization, taxa with relative abundance <1% are grouped as “Others” in the graph).

O-D and H-D samples were annotated to 126 and 135 viral species, respectively (Figure 10, Table S4). Among them, three pooled samples from each region shared 48 species in O-D and 37 species in H-D. Additionally, O-D-FA-MIX and O-D-EL-MIX were co-annotated with 10 species; O-D-FA-MIX and O-D-SA-MIX were co-annotated with 4 species; and O-D-EL-MIX and O-D-SA-MIX were co-annotated with 7 species.

H-D-FA-MIX and H-D-SA-MIX were co-annotated with 18 species; H-D-EL-MIX and H-D-SA-MIX were co-annotated with 29 species. Notably, 29 species were co-annotated across all six samples. Among them, Dickeya phage phiDP23.1 accounted for the highest relative abundance, with an average proportion of 59.60%. Additionally, African swine fever virus (ASFV) was annotated in both H-D-EL-MIXs and H-D-SA-MIX.

## 4. Discussion

In the 16S rRNA high-throughput sequencing results, a higher Shannon index and lower Simpson index indicated higher microbial community diversity, whereas a higher ACE value reflected a larger estimated total number of species [44,45]. In this study, these indices varied across samples, indicating inconsistent bacterial diversity and species richness among them. Based on these estimators, the sequencing data of this study showed that at the phylum level, except for the dominant phylum of H-D-FA being Firmicutes, Proteobacteria was the dominant phylum in 13 of the 14 samples, with the exception of H-D-FA, wherein Firmicutes was predominant. This finding differs from previous reports that consistently identified Proteobacteria as the dominant phylum in ticks [38,44,48,49,50,51,52,53,54]. Moreover, although the total number of annotated phyla varied between O-D and H-D, the co-annotated phyla across all samples were consistent. This observation is consistent with those of prior studies that identified four dominant phyla in *D. nuttalli* [38,44,48].

At the genus level, although the dominant genera varied between O-D and H-D at different growth stages, there were some similarities. The total relative abundance of *Rickettsia* was the highest in H-D and second highest in O-D. The relative abundance of *Rickettsia* in O-D and H-D exhibited an alternating trend of increase and decrease from first-generation adult female ticks to second-generation adult ticks, with the highest abundance observed in O-D-E and H-D-E. Notably, the abundance of *Rickettsia* increased significantly during the two blood-feeding stages, namely from larvae to engorged larvae and from nymphs to engorged nymphs. This trend is consistent with previous results reported in *D. nuttalli* [44] and supports the general observation that blood-feeding behavior affects the composition and abundance of the microbiome in ticks [55,56]. However, it has also been reported that blood feeding reduces microbial richness and internal tick microbiota diversity [44], suggesting that the process of blood ingestion affects the tick microbiome. *Rickettsia*, as one of the symbionts of ticks, can provide ticks with B vitamins such as folic acid [57]. Pathogenic *Rickettsia* can modulate the immune response of ticks [58]. In a previous study, multiple *Rickettsia* species were detected in *D. nuttalli*; therefore, it is a serious threat to human and animal health worldwide [44] and requires increased attention.

*Staphylococcus* was one of the dominant genera in O-D-FA and H-D-FA. However, its abundance was relatively low in the other six developmental stages. *Staphylococcus* can cause skin infections, food poisoning, and sepsis in both humans and animals [59]. Moreover, *Staphylococcus* is the dominant genus on the skin near the site of tick bites [60]. *Staphylococcus* is resistant to various antibiotics, and the treatment of related infections is complex and difficult [61]. In this study, the high abundance of *Staphylococcus* may be attributed to the host.

Furthermore, it is worth noting that *Coxiella* showed high abundance in O-D-E and H-D-L. *Coxiella* is regarded as an endosymbiont genus that can be propagated through tansovarian and transstadial transmission [62,63]. *Coxiella* is primarily enriched in the midgut, ovaries, and Malpighian tubules of ticks and plays a significant role in tick reproduction and development [64]. The removal of *Coxiella*-like endosymbionts can reduce the satiation weight of *Amblyomma americanum* and *H. longicornis* and the egg-laying ability of adult ticks [64,65]. This may explain the high abundance of *Coxiella* in O-D-E and H-D-L in this study.

Further, we annotated various *Pseudomonas* speices across all developmental stages of O-D and H-D. *P. aeruginosa* has also been detected in *H. flava* [66], *Rhipicehalus microplus* [67], and *Dermacentor variabilis* [68]. *P. aeruginosa* was first isolated from purulent wound secretions [69]. *P. aeruginosa* poses a serious threat to animal health and can lead to significant economic losses in the livestock industry. Therefore, more attention should be focused on the control of *D. nuttalli* infestations.

Viruses are an important component of tick-borne pathogens. According to previous reports, 19 tick-borne viruses have been identified in China [3], most of which are transmitted by hard ticks [70]. In this study, four common viral orders were detected in O-D and H-D samples, whereas Bunyavirales and Picornavirale were uniquely identified in H-D. Almost all studies on tick viruses have detected viruses associated with existing members of Bunyavirales [71]. Most of them belong to the Nairoviridae and Phenuiviridae families, which usually infect vertebrates and pose a threat to human and animal health [46]. At the family level, 25 and 28 viral families were annotated in O-D and H-D, respectively, with 11 viral families common to both. The family Mimiviridae belongs to a class of nucleocytoplasmic large DNA viruses, which are giant viruses that infect amoebae [46]. At the species level, Pestivirus A (bovine viral diarrhea virus 1, BVDV-1) was identified at notable abundance in all six samples. BVDV-1 primarily affects cattle and can lead to high mortality in severe cases [72]. Moreover, it is associated with persistent infection and immunosuppression in affected cattle [73]. In this study, Tacheng Tick Virus 2 (TcTV-2), a member of the genus *Phlebovirus,* was annotated in H-D-FA-MIX and H-D-SA-MIX. TcTV-2 was first discovered through next-generation sequencing of ticks in China [74]. To date, the pathogenic potential of TcTV-2 infection remains unclear, with only one human case reported in China in 2019 [75]. Interestingly, ASFV was detected in H-D-EL-MIX and H-D-SA-MIX but not in H-D-FA-MIX. ASFV was first reported in Asia in 2018 in Liaoning Province, China [76], and rapidly spread throughout mainland China [77]. Although ticks of the genus *Ornithodoros* are the biological vectors for ASFV, DNA fragments of this virus have also been detected in hard ticks, including *D. nuttalli* [78,79]. However, there is currently no conclusive evidence to suggest that hard ticks can transmit ASFV [80].

The microbial community composition and diversity of ticks are affected by several factors, including tick species and hosts [81], sex [82], developmental stage [83], blood meal [84], engorged state [85,86], and geographical location [87]. In this study, the two *D. nuttalli* sampling locations were >1500 km apart. These regions differ significantly in average temperature, precipitation, and sunshine duration, which may partly explain the observed differences in the diversity and abundance of microorganisms carried by *D. nuttalli*. Overall, this study compared the microorganisms of *D. nuttalli* from different regions in Inner Mongolia and analyzed the differences in the spectrum of pathogens in *D. nuttalli*. Our findings may help predict emerging pathogens and evaluate the potential risks to both animal and public health.

## 5. Conclusions

In both O-D and H-D samples, Proteobacteria was the phylum with the highest total abundance. Notable differences in dominant bacterial genera were observed across different developmental stages in O-D and H-D. To our knowledge, this study is the first to compare the composition of the microbial community across the developmental stages of *D. nuttalli* from two locations in Inner Mongolia under identical artificial feeding conditions. Moreover, for the first time, microorganisms such as *R. japonica*, TcTV-2, and BVDV were annotated in *D. nuttalli* with certain abundance.

## Figures and Tables

**Table 1:** Sequencing data of *Dermacentor nuttalli* samples from Ordos and Hinggan League.

Region	Sample	Sequences	Bases (bp)	Average Length (bp)
Ordos	O-D-EN	41,334	17,437,036	421.86
	O-D-EL	255,821	107,654,007	420.82
	O-D-SA	62,405	26,458,024	423.97
	O-D-E	33,565	14,034,524	418.13
	O-D-N	59,702	25,172,642	421.64
	O-D-FA	36,789	15,430,339	419.43
	O-D-L	73,069	30,818,614	421.77
Hinggan League	H-D-EN	30,495	12,936,993	424.23
	H-D-EL	28,435	11,635,803	409.21
	H-D-SA	131,101	55,929,480	426.61
	H-D-E	60,090	24,652,783	410.26
	H-D-N	80,153	33,865,155	422.51
	H-D-FA	68,050	28,708,458	421.87
	H-D-L	36,736	15,595,860	424.54

Note: O-D-FA: first-generation adult female ticks from Ordos, O-D-E: eggs from Ordos, O-D-L: larvae from Ordos, O-D-EL: engorged larvae from Ordos, O-D-N: nymphs from Ordos, O-D-EN: engorged nymphs from Ordos, O-D-SA: second-generation adult ticks from Ordos. H-D-FA: first-generation adult female ticks from Hinggan League, H-D-E: eggs from Hinggan League, H-D-L: larvae from Hinggan League, H-D-EL: engorged larvae from Hinggan League, H-D-N: nymphs from Hinggan League, H-D-EN: engorged nymphs from Hinggan League, H-D-SA: second-generation adult ticks from Hinggan League.

**Table 2:** Alpha diversity indices of *Dermacentor nuttalli* from two regions.

Region	Sample ID	Reads	0.97					
			OTU	Ace	Chao	Coverage	Shannon	Simpson
Ordos	O-D-FA	27,346	74	94	81	0.999525	2.46	0.1424
	O-D-E	30,080	68	84	83	0.999501	1.28	0.3973
	O-D-L	57,407	109	113	113	0.999843	2.58	0.1732
	O-D-EL	245,845	165	171	169	0.999943	1.17	0.467
	O-D-N	52,228	89	102	101	0.999713	1.96	0.2667
	O-D-EN	37,595	55	61	59	0.999787	1.61	0.3281
	O-D-SA	53,541	100	107	103	0.999813	2.19	0.1792
Hinggan League	H-D-FA	60,335	75	81	81	0.999834	1.77	0.2695
	H-D-E	55,182	68	77	75	0.999783	0.88	0.6072
	H-D-L	32,873	123	139	132	0.999331	1.81	0.2977
	H-D-EL	25,539	100	109	107	0.999452	1.52	0.4618
	H-D-N	66,081	84	94	95	0.999818	2.81	0.0814
	H-D-EN	21,434	99	108	106	0.999347	2.38	0.1461
	H-D-SA	113,346	71	79	76	0.999912	1.99	0.1888

Note: O-D-FA: first-generation adult female ticks from Ordos, O-D-E: eggs from Ordos, O-D-L: larvae from Ordos, O-D-EL: engorged larvae from Ordos, O-D-N: nymphs from Ordos, O-D-EN: engorged nymphs from Ordos, O-D-SA: second-generation adult ticks from Ordos. H-D-FA: first-generation adult female ticks from Hinggan League, H-D-E: eggs from Hinggan League, H-D-L: larvae from Hinggan League, H-D-EL: engorged larvae from Hinggan League, H-D-N: nymphs from Hinggan League, H-D-EN: engorged nymphs from Hinggan League, H-D-SA: second-generation adult ticks from Hinggan League.

**Table 3:** Relative abundance of bacterial phyla in different developmental stages of *Dermacentor nuttalli* from Ordos and Hinggan League.

Region	Sample	Proteobacteria (%)	Actinobacteria (%)	Firmicutes (%)	Bacteroidetes (%)	Other (%)
Ordos	O-D-FA	78.81	3.19	16.55	1.42	0.03
	O-D-E	94.56	0.35	1.32	3.76	—
	O-D-L	81.91	11.00	3.04	4.04	—
	O-D-EL	93.86	4.11	1.98	0.03	0.02
	O-D-N	78.32	19.85	0.90	0.94	—
	O-D-EN	96.49	1.99	1.47	0.05	—
	O-D-SA	93.13	5.49	0.61	0.76	0.01
	Total proportion	88.16	6.57	3.69	1.57	0.01
Hinggan League	H-D-FA	45.64	53.70	0.21	0.44	—
	H-D-E	99.43	0.30	0.08	0.18	—
	H-D-L	91.42	2.39	0.37	5.71	0.11
	H-D-EL	93.17	2.78	0.33	3.62	0.11
	H-D-N	81.74	1.07	5.13	12.06	—
	H-D-EN	92.57	4.10	0.31	2.98	0.04
	H-D-SA	68.17	0.68	30.82	0.33	—
	Total proportion	81.73	9.29	5.32	3.62	0.04

Note: O-D-FA: first-generation adult female ticks from Ordos, O-D-E: eggs from Ordos, O-D-L: larvae from Ordos, O-D-EL: engorged larvae from Ordos, O-D-N: nymphs from Ordos, O-D-EN: engorged nymphs from Ordos, O-D-SA: second-generation adult ticks from Ordos. H-D-FA: first-generation adult female ticks from Hinggan League, H-D-E: eggs from Hinggan League, H-D-L: larvae from Hinggan League, H-D-EL: engorged larvae from Hinggan League, H-D-N: nymphs from Hinggan League, H-D-EN: engorged nymphs from Hinggan League, H-D-SA: second-generation adult ticks from Hinggan League. — represents no annotation.

**Table 4:** Statistical data of viral taxa in *Dermacentor nuttalli* samples.

Region	Sample	Kingdom	Phylum	Class	Order	Family	Genus	Species
Ordos	O-D-FA-MIX	norank	norank	norank	4	24	48	77
	O-D-EL-MIX	norank	norank	norank	5	22	49	75
	O-D-SA-MIX	norank	norank	norank	4	18	47	91
	Total	-	-	-	4	26	61	126
Hinggan League	H-D-FA-MIX	norank	norank	norank	5	18	35	61
	H-D-EL-MIX	norank	norank	norank	5	20	42	66
	H-D-SA-MIX	norank	norank	norank	6	28	49	129
	Total	-	-	-	6	28	49	135

Note: O-D-FA-MIX: first-generation adult female ticks from Ordos; O-D-EL-MIX: mixed samples of eggs and larvae from Ordos; O-D-SA-MIX: second-generation adult ticks from Ordos; H-D-FA-MIX: first-generation adult female ticks from Hinggan League; H-D-EL-MIX: mixed samples of eggs and larvae from Hinggan League; H-D-SA: second-generation adult ticks from Hinggan League.

## Data Availability

The raw tags were deposited in Sequence Read Archive (SRA) from the NCBI under BioProject accession number PRJNA1222544 and PRJNA1222561. The individual run files received the accession numbers SAMN46783900–SAMN46783913 and SAMN46784101–SAMN46784106.

## Supplementary Materials

The following supporting information can be downloaded at https://www.mdpi.com/article/10.3390/biology14060613/s1. Table S1. Genus-level microbial community composition of *Dermacentor nuttalli* at different developmental stages in Ordos and Hinggan League. Table S2. Species-level microbial community composition of *Dermacentor nuttalli* at different developmental stages in Ordos and Hinggan League. Table S3. Viral genus data of *Dermacentor nuttalli*. Table S4. Viral species data of *Dermacentor nuttalli*.

## Abbreviations

The following abbreviations are used in this manuscript:

PCR	polymerase chain reaction
OTUs	operational taxonomic units.
ACE	abundance-based coverage estimator
