# Supplementary material for: Comparative Analysis of Microbial Communities in Each Developmental Stage of Dermacentor nuttalli in Two Regions in Inner Mongolia, China
Source: Biology (Basel). 2025 May 27;14(6):613. doi: 10.3390/biology14060613 (PMC12189743; doi:10.3390/biology14060613)
Supplement: Supplementary file 1 [file biology-14-00613-s001.zip › biology-3618930-supplementary.pdf]

Table S1. Genus-level microbial community composition of *Dermacentor nuttalli* at different developmental stages in Ordos and Hinggan League.

**Region**	Bacterial species	Relative abundance (%)							
		First-generation female adult ticks	Eggs	Larval ticks	Engorged larval ticks	Nymph ticks	Engorged nymph ticks	Second-generation adult ticks	Total abundance
**Ordos**	Arsenophonus	0.11	1.21	2.14	63.72	8.32	52.63	0.03	18.31
	Rickettsia	30.69	44.19	2.30	24.38	4.17	7.29	11.43	17.78
	Pseudomonas	11.42	1.32	5.42	0.03	47.58	5.10	31.03	14.56
	Coxiella	3.72	44.84	0.40	0.25	1.23	0.11	30.19	11.53
	Stenotrophomonas	1.66	1.00	41.78	0.11	3.05	6.45	6.26	8.62
	Brevibacterium	0.19	0.13	4.72	0.99	16.45	1.45	3.93	3.98
	Ac37b	0.20	0.03	0.21	4.40	1.01	20.23	0.00	3.72
	Staphylococcus	16.53	1.25	2.72	0.65	0.82	1.16	0.43	3.36
	Brevundimonas	0.99	0.13	9.93	0.38	3.89	0.14	3.06	2.65
	Brucella	0.03	0.05	6.93	0.06	4.99	0.10	1.33	1.93
	Anaplasma	13.27	0.03	0.03	0.00	0.02	0.00	0.04	1.91
	Alcaligenaceae_unclassified	5.50	0.02	2.55	0.01	1.17	0.12	0.15	1.36
	Rhodococcus	0.56	0.15	3.77	0.33	2.59	0.29	0.42	1.16
	Delftia	0.12	0.04	4.71	0.01	1.67	0.78	0.24	1.08
	Serratia	5.01	0.01	2.04	0.00	0.01	0.01	0.03	1.02
	Enterobacter	1.91	0.20	0.61	0.05	0.04	0.01	4.00	0.97
	Sphingobacterium	0.62	0.88	2.73	0.02	0.72	0.04	0.68	0.81
	Raoultella	3.59	0.01	0.35	0.00	0.01	0.00	0.01	0.57
	Alcaligenes	0.00	0.22	0.08	0.00	0.05	0.00	3.36	0.53
	Flavobacterium	0.73	2.72	0.04	0.00	0.03	0.00	0.00	0.50
	Acinetobacter	0.28	0.02	0.04	0.00	0.06	2.86	0.23	0.50
	Tsukamurella	0.00	0.00	0.03	2.19	0.00	0.00	0.00	0.32
	Glutamicibacter	1.79	0.04	0.03	0.00	0.00	0.05	0.01	0.27
	Enterococcus	0.00	0.00	0.01	1.18	0.01	0.00	0.00	0.17

	Others	1.11	1.52	6.44	1.24	2.11	1.18	3.12	2.39
Hinggan League	Rickettsia	28.16	76.15	11.37	66.76	1.50	17.25	0.65	28.83
	Stenotrophomonas	4.90	16.79	13.66	1.54	18.84	23.92	0.13	11.40
	Pseudomonas	7.56	0.83	9.77	1.86	15.19	27.12	16.19	11.22
	Coxiella	1.00	1.51	50.97	1.41	5.91	0.67	0.22	8.81
	Staphylococcus	43.62	0.23	2.23	2.40	0.98	2.20	0.43	7.44
	Alcaligenes	0.11	0.01	0.00	0.02	0.00	0.53	33.38	4.86
	Myroides	0.07	0.00	0.03	0.00	0.00	0.10	30.56	4.40
	Delftia	0.08	0.19	0.20	0.88	8.12	6.48	0.01	2.28
	Acinetobacter	0.05	0.14	0.28	6.27	1.71	6.89	0.00	2.19
	Enterobacter	0.08	3.06	0.19	0.13	11.67	0.07	0.02	2.17
	Brevundimonas	0.55	0.17	0.53	0.57	9.85	1.54	1.33	2.08
	Ac37b	0.00	0.01	0.00	11.96	0.11	0.50	0.00	1.80
	Enterococcus	10.06	0.06	0.05	0.14	0.01	0.33	0.07	1.53
	Brevibacterium	0.02	0.07	0.17	2.25	4.19	2.36	0.19	1.32
	Providencia	0.03	0.00	0.00	0.00	0.00	0.06	7.43	1.07
	Ochrobactrum	0.17	0.08	0.30	0.52	6.02	0.25	0.07	1.06
	Comamonas	0.00	0.00	0.03	0.02	1.28	5.42	0.00	0.96
	Leucobacter	0.02	0.01	0.02	0.06	6.30	0.07	0.06	0.94
	Achromobacter	1.69	0.07	3.57	0.09	0.56	0.17	0.05	0.88
	Paenalcaligenes	0.01	0.00	0.00	0.01	0.00	0.04	5.38	0.78
	Rhodococcus	0.22	0.07	4.11	0.16	0.33	0.21	0.03	0.73
	Sphingobacterium	0.07	0.06	0.09	0.15	4.15	0.12	0.24	0.70
	Proteus	0.00	0.00	0.00	0.00	0.00	0.04	3.18	0.46
	Aerococcus	0.00	0.00	0.00	0.01	0.02	1.31	0.00	0.19
	Others	1.50	0.49	2.44	2.78	3.27	2.37	0.35	1.88

Note: To make the view best, the part with a relative abundance of less than 1% is merged into others and displayed in the graph.

Table S2. Species-level microbial community composition of *Dermacentor nuttalli* at different developmental stages in Ordos and Hinggan League.

Region	Bacterial species	Relative abundance (%)							
		First-generation female adult ticks	Eggs	Larval ticks	Engorged larval ticks	Nymph ticks	Engorged nymph ticks	Second-generation adult ticks	Total abundance
Ordos	Arsenophonus_uncultured_bacterium	0.11	1.21	2.14	63.72	8.32	52.63	0.03	18.31
	Rickettsia_japonica	30.69	44.19	2.30	24.38	4.17	7.29	11.43	17.78
	Coxiella_uncultured_coxiella_sp.	3.72	44.84	0.40	0.25	1.23	0.11	30.19	11.53
	Pseudomonas_aeruginosa	0.23	0.66	0.08	0.00	47.42	4.02	24.70	11.02
	[Pseudomonas]_geniculata	1.14	0.17	38.49	0.10	2.61	6.40	6.17	7.87
	Brevibacterium_epidermidis	0.19	0.13	4.71	0.99	16.45	1.45	3.93	3.98
	Rickettsiales_bacterium_Ac37b	0.20	0.03	0.21	4.40	1.01	20.23	0.00	3.72
	Brevundimonas_diminuta	0.99	0.12	9.68	0.11	3.86	0.14	3.06	2.57
	Staphylococcus_sciuri	11.14	0.83	2.42	0.54	0.76	1.14	0.37	2.46
	Pseudomonas_unclassified	4.10	0.39	4.32	0.01	0.14	1.08	6.16	2.31
	Brucella_unclassified	0.03	0.05	6.93	0.06	4.99	0.10	1.33	1.93
	Anaplasma_marginale	13.27	0.03	0.03	0.00	0.02	0.00	0.04	1.91
	Alcaligenaceae_unclassified	5.50	0.02	2.55	0.01	1.17	0.12	0.15	1.36
	Rhodococcus_erythropolis	0.56	0.15	3.76	0.33	2.59	0.29	0.42	1.16
	Pseudomonas_azotoformans	6.75	0.26	0.98	0.01	0.02	0.00	0.07	1.16
	Delftia_tsuruhatensis	0.12	0.04	4.71	0.01	1.67	0.78	0.24	1.08
	Serratia_unclassified	4.97	0.01	2.03	0.00	0.01	0.01	0.01	1.01
	Enterobacter_unclassified	1.91	0.20	0.61	0.05	0.04	0.01	4.00	0.97
	Staphylococcus_hyicus	5.22	0.33	0.01	0.00	0.03	0.00	0.04	0.81
	Stenotrophomonas_nitritireducens	0.48	0.29	3.29	0.01	0.43	0.05	0.09	0.66
	Raoultella_planticola	3.59	0.01	0.35	0.00	0.01	0.00	0.01	0.57
	Alcaligenes_unclassified	0.00	0.22	0.08	0.00	0.05	0.00	3.36	0.53

	Flavobacterium_ummariense	0.73	2.72	0.03	0.00	0.02	0.00	0.00	0.50
	Acinetobacter_unclassified	0.05	0.00	0.03	0.00	0.06	2.86	0.08	0.44
	Tsukamurella_unclassified	0.00	0.00	0.03	2.19	0.00	0.00	0.00	0.32
	Glutamicibacter_arilaitensis	1.79	0.04	0.03	0.00	0.00	0.05	0.01	0.27
	Sphingobacterium_siyangense	0.00	0.00	1.14	0.00	0.10	0.02	0.03	0.18
	Enterococcus_faecalis	0.00	0.00	0.01	1.18	0.01	0.00	0.00	0.17
	Others	2.55	3.07	8.64	1.63	2.79	1.21	4.06	3.42
Hinggan League	Rickettsia_japonica	28.16	76.15	11.37	66.76	1.50	17.25	0.65	28.83
	Coxiella_uncultured_coxiella_sp.	1.00	1.51	50.97	1.41	5.91	0.67	0.22	8.81
	Pseudomonas_aeruginosa	0.35	0.08	0.01	1.19	5.25	21.57	16.07	6.36
	Staphylococcus_hyicus	41.60	0.14	0.10	0.85	0.00	0.09	0.19	6.14
	Alcaligenes_unclassified	0.11	0.01	0.00	0.02	0.00	0.53	33.38	4.86
	Stenotrophomonas_unclassified	1.05	0.15	0.47	0.61	2.64	23.52	0.01	4.06
	Stenotrophomonas_sp._MYb57	3.37	0.34	0.85	0.80	16.05	0.24	0.11	3.11
	Pseudomonas_unclassified	1.77	0.55	0.46	0.53	9.93	5.38	0.09	2.67
	Myroides_odoratimimus	0.03	0.00	0.01	0.00	0.00	0.06	18.19	2.61
	Stenotrophomonas_rhizophila	0.05	16.24	0.12	0.11	0.03	0.01	0.00	2.37
	Delftia_tsuruhatensis	0.08	0.19	0.20	0.88	8.12	6.48	0.01	2.28
	Pseudomonas_azotoformans	5.44	0.20	9.29	0.14	0.00	0.16	0.03	2.18
	Enterobacter_unclassified	0.08	3.06	0.19	0.13	11.67	0.07	0.02	2.17
	Brevundimonas_diminuta	0.51	0.17	0.49	0.54	9.85	1.54	1.33	2.06
	Stenotrophomonas_nitritireducens	0.43	0.06	12.22	0.03	0.12	0.15	0.00	1.86
	Rickettsiales_bacterium_Ac37b	0.00	0.01	0.00	11.96	0.11	0.50	0.00	1.80
	Enterococcus_faecium	10.06	0.06	0.05	0.14	0.01	0.33	0.07	1.53
	Brevibacterium_epidermidis	0.02	0.07	0.13	2.25	4.19	2.36	0.19	1.32

Acinetobacter_unclassified	0.01	0.03	0.24	5.16	1.67	1.64	0.00	1.25
Staphylococcus_sciuri	1.92	0.08	2.00	1.48	0.65	2.06	0.25	1.21
Myroides_odoratus	0.03	0.00	0.00	0.00	0.00	0.03	8.32	1.20
Providencia_rettgeri	0.03	0.00	0.00	0.00	0.00	0.06	7.43	1.07
Ochrobactrum_pseudogrignonense	0.17	0.08	0.30	0.52	6.02	0.25	0.07	1.06
Acinetobacter_pittii	0.01	0.10	0.04	1.07	0.01	5.24	0.00	0.93
Achromobacter_unclassified	1.69	0.07	3.57	0.09	0.56	0.17	0.05	0.88
Alcaligenes_faecalis	0.01	0.00	0.00	0.00	0.00	0.04	5.38	0.78
Comamonas_testosteroni	0.00	0.00	0.00	0.00	0.00	5.42	0.00	0.78
Leucobacter_uncultured_bacterium	0.02	0.00	0.02	0.03	5.06	0.07	0.06	0.75
Rhodococcus_erythropolis	0.22	0.07	3.99	0.16	0.33	0.21	0.03	0.72
Myroides_sp._ZB35	0.01	0.00	0.00	0.00	0.00	0.01	4.05	0.58
Proteus_vulgaris	0.00	0.00	0.00	0.00	0.00	0.04	3.18	0.46
Sphingobacterium_multivorum	0.02	0.02	0.07	0.07	2.41	0.00	0.00	0.37
Sphingobacterium_faecium	0.03	0.03	0.00	0.01	1.60	0.01	0.00	0.24
Aerococcus_urinaeequi	0.00	0.00	0.00	0.01	0.02	1.31	0.00	0.19
Comamonas_koreensis	0.00	0.00	0.00	0.01	1.28	0.00	0.00	0.19
Leucobacter_unclassified	0.00	0.00	0.00	0.03	1.24	0.00	0.00	0.18
Others	1.67	0.52	2.82	2.99	3.77	2.54	0.59	2.13

Note: To make the view best, the part with a relative abundance of less than 1% is merged into others and displayed in the graph.

Table S3. Virus genus data of *Dermacentor nuttalli*.

Taxon	O-D-FA-MIX	O-D-EL-MIX	O-D-SA-MIX	H-D-FA-MIX	H-D-EL-MIX	H-D-SA-MIX
Limestonevirus	0.949201687	0.544168770	0.505499654	0.425067593	0.366277777	0.785591552
Gammaretrovirus	0.002502053	0.108287324	0.087027952	0.075423182	0.263440630	0.036672087
norank_d__Viruses	0.011439168	0.033063230	0.089623158	0.056062034	0.018283257	0.048304541
Tupanvirus	0.000986729	0.019889529	0.030569617	0.047401994	0.019655443	0.014137001
Avipoxvirus	0.002294207	0.059849139	0.069020585	0.029680719	0.030732427	0.019667415
norank_f__Phycodnaviridae	0.000645147	0.026519368	0.024794186	0.060884000	0.034611965	0.005831784
norank_f__Myoviridae	0.013742500	0.004334573	0.008544328	0.006163063	0.001200324	0.012289594
Prasinovirus	0.002561095	0.022980937	0.010441632	0.016957419	0.037423455	0.018347847
Klosneuvirus	0.000654466	0.007073274	0.011553663	0.014229788	0.007709810	0.002361088
Bracovirus	0.000000000	0.000000000	0.001166174	0.009320830	0.000890484	0.000000000
Errantivirus	0.000000000	0.001370015	0.008607325	0.015584591	0.031388663	0.000000000
Indivirus	0.000583391	0.026429944	0.019027353	0.018190245	0.007363458	0.007059675
Hokovirus	0.000400049	0.002597800	0.009307888	0.025959414	0.008864963	0.000259290
norank_f__Poxviridae	0.000087267	0.008644441	0.010621086	0.025597492	0.003324031	0.001143709
norank_f__Mimiviridae	0.001190302	0.004362019	0.000490905	0.010230243	0.003019456	0.000000000
norank_f__Siphoviridae	0.003476561	0.001189884	0.000721644	0.003176877	0.000000000	0.000320959
Marseillevirus	0.000110449	0.012163614	0.007355047	0.004718401	0.013804103	0.000000000
Pandoravirus	0.000164489	0.000304668	0.002234866	0.005683082	0.022474252	0.000446447
Pestivirus	0.000396837	0.010818927	0.008421906	0.004930003	0.005727036	0.002609261
Catovirus	0.000361575	0.012020857	0.002728808	0.012493204	0.011202670	0.000163800
Cafeteriavirus	0.000767938	0.000545542	0.001707097	0.009462676	0.000000000	0.000357695
norank_f__Luteoviridae	0.000000000	0.000245317	0.000000000	0.000000000	0.000000000	0.000000000
Chlorovirus	0.000037140	0.002283655	0.002495983	0.003786376	0.000676108	0.000000000
Orthohepevirus	0.000295335	0.010278416	0.008121527	0.001762670	0.000000000	0.004765308

Lymphocystivirus	0.000598472	0.017468329	0.007564000	0.008381029	0.002173661	0.004143987
Cyprinivirus	0.000257842	0.013631127	0.021890480	0.009112777	0.004772530	0.007490531
Mimivirus	0.000156619	0.007373733	0.003938022	0.007338362	0.002715933	0.003695957
Alpharetrovirus	0.000184119	0.003718234	0.002524469	0.005075470	0.000000000	0.005386394
Badnavirus	0.000000000	0.000503718	0.000957398	0.010664509	0.037053042	0.000000000
Macavirus	0.000000000	0.000685330	0.000474653	0.002105214	0.000662310	0.001211784
Iridovirus	0.000019376	0.000634938	0.000246769	0.001303451	0.000000000	0.000000000
Toursvirus	0.000000000	0.001412292	0.001268618	0.001982584	0.000000000	0.000359863
norank_f__Iridoviridae	0.000045138	0.003652134	0.000000000	0.003555939	0.017214572	0.000000000
Betaentomopoxvirus	0.000000000	0.000000000	0.000000000	0.001996558	0.003768760	0.000000000
Others	0.006840049	0.031498921	0.041053207	0.065718210	0.043568879	0.017382433

Note: O-D-FA-MIX: first-generation adult ticks of Ordos; O-D-EL-MI: mixed samples of eggs and larval ticks of Ordos; O-D-SA-MIXX: second-generation adult ticks of Ordos; H-D-FA-MIX: first-generation adult ticks of Hinggan League; H-D-EL-MIX: mixed samples of eggs and larval ticks of Hinggan League; H-D-SA-MIX: second-generation adult ticks of Hinggan League. To make the view best, the part with a relative abundance of less than 1% is merged into others and displayed in the graph.

Table S4. Virus species data of *Dermacentor nuttalli*.

	O-D-FA-MIX	O-D-EL-MIX	O-D-SA-MIX	H-D-FA-MIX	H-D-EL-MIX	H-D-SA-MIX
Dickeya_phage_phiDP23.1	0.949201687	0.544168770	0.505499654	0.425067593	0.366277777	0.785591552
Feline_leukemia_virus	0.000924308	0.035551340	0.045743730	0.031155125	0.020889935	0.025147518
uncultured_virus	0.002813396	0.012816610	0.073402109	0.031448694	0.005848586	0.006060296
Flamingopox_virus_FGPVKD09	0.001364744	0.043449033	0.046378816	0.024237877	0.028835811	0.013223357
Aureococcus_anophagefferens_virus	0.000553659	0.024626215	0.020551582	0.042428295	0.015019077	0.004865086
Tupanvirus_soda_lake	0.000580131	0.013250624	0.018290029	0.020188306	0.013507296	0.003205341
Trichoplusia_ni_TED_virus	0.000000000	0.001370015	0.008607325	0.015584591	0.031388663	0.000000000
Tupanvirus_deep_ocean	0.000406598	0.006638905	0.012279588	0.027213688	0.006148148	0.010931660
Murine_osteosarcoma_virus	0.000660762	0.021187979	0.027430837	0.018896746	0.004295277	0.008930068

Indivirus_ILV1	0.000583391	0.026429944	0.019027353	0.018190245	0.007363458	0.007059675
Hokovirus_HKV1	0.000400049	0.002597800	0.009307888	0.025959414	0.008864963	0.000259290
Klosneuvirus_KNV1	0.000616407	0.002812724	0.006676071	0.003960754	0.003861242	0.000954621
Diachasmimorpha_entomopoxvirus	0.000087267	0.008644441	0.010621086	0.025597492	0.003324031	0.001143709
Brazilian_marseillevirus	0.000092185	0.006178851	0.006717356	0.004463066	0.013804103	0.000000000
Cotesia_plutellae_polydnavirus	0.000000000	0.000000000	0.000000000	0.003099624	0.000000000	0.000000000
Canarypox_virus	0.000929463	0.016400107	0.021182000	0.005134652	0.001896616	0.006444058
Pestivirus_A	0.000396837	0.010818927	0.008421906	0.004930003	0.005727036	0.002609261
Chrysochromulina_ericina_virus	0.000051961	0.001263506	0.002359190	0.013096403	0.014293145	0.000401199
Dishui_lake_phycodnavirus_1	0.000309682	0.008824249	0.004583693	0.005173886	0.022274131	0.003933539
Catovirus_CTV1	0.000361575	0.012020857	0.002728808	0.012493204	0.011202670	0.000163800
Cafeteria_roenbergensis_virus	0.000767938	0.000545542	0.001707097	0.009462676	0.000000000	0.000357695
Porcine_type-C_oncovirus	0.000000000	0.020342440	0.011147325	0.014051630	0.234037549	0.000761249
Orthohepevirus_A	0.000295335	0.010278416	0.008121527	0.001762670	0.000000000	0.004765308
Norway_luteo-like_virus_2	0.000000000	0.000245317	0.000000000	0.000000000	0.000000000	0.000000000
Bodo_saltans_virus	0.000038059	0.004260550	0.004877592	0.010269034	0.003848568	0.001406467
Murine_leukemia_virus	0.000870016	0.030412309	0.002009042	0.005944682	0.001875466	0.001209868
Pandoravirus_neocaledonia	0.000000000	0.000000000	0.001932203	0.000309466	0.021029678	0.000000000
Anguillid_herpesvirus_1	0.000257842	0.013631127	0.021890480	0.009112777	0.004772530	0.007490531
Acanthamoeba_polyphaga_mimivirus	0.000156619	0.007373733	0.003938022	0.007338362	0.002715933	0.003695957
Diolcogaster_facetosa_bracovirus	0.000000000	0.000000000	0.000146158	0.000000000	0.000000000	0.000000000
Lymphocystis_disease_virus_-_isolate_China	0.000367043	0.007709963	0.003286060	0.005623787	0.000000000	0.001514729
Cotesia_sesamiae_bracovirus	0.000000000	0.000000000	0.000000000	0.004288322	0.000000000	0.000000000
Bovine_gammaherpesvirus_6	0.000000000	0.000685330	0.000474653	0.002105214	0.000662310	0.001211784
Namao_virus	0.000067486	0.003780807	0.000490905	0.004077248	0.000772695	0.000000000
Erysipelothrix_phage_phi1605	0.000000000	0.000000000	0.000721644	0.002973815	0.000000000	0.000000000
Echidna_ERV	0.000000000	0.000000000	0.000000000	0.000000000	0.000000000	0.000000000

Diadromus_pulchellus_toursvirus	0.000000000	0.001412292	0.001268618	0.001982584	0.000000000	0.000359863
uncultured_Mediterranean_phage_uvMED	0.000282843	0.000000000	0.000000000	0.000000000	0.000000000	0.029882741
Invertebrate_iridescent_virus_31	0.000000000	0.000000000	0.000246769	0.000000000	0.000000000	0.000000000
Ixodes_scapularis_associated_virus_1	0.000000000	0.000000000	0.000000000	0.000000000	0.000000000	0.000000000
Cacao_swollen_shoot_Togo_A_virus	0.000000000	0.000000000	0.000000000	0.005526187	0.027817034	0.000000000
Anopheles_minimus_irodovirus	0.000045138	0.003652134	0.000000000	0.002708859	0.016148591	0.000000000
Others	0.036517581	0.096619143	0.087932886	0.154143028	0.101497679	0.066419778

Note: O-D-FA-MIX: first-generation adult ticks of Ordos; O-D-EL-MIX: mixed samples of eggs and larval ticks of Ordos; O-D-SA-MIX: second-generation adult ticks of Ordos; H-D-FA-MIX: first-generation adult ticks of Hinggan League; H-D-EL-MIX: mixed samples of eggs and larval ticks of Hinggan League; H-D-SA-MIX: second-generation adult ticks of Hinggan League. To make the view best, the part with a relative abundance of less than 1% is merged into others and displayed in the graph.
